# Quantitative evaluation of essential amino acids and omega-3 long-chain polyunsaturated fatty acids from global marine bivalve aquaculture

**DOI:** 10.1016/j.fochx.2025.102181

**Published:** 2025-01-23

**Authors:** Karsoon Tan, Peng Xu, Leiheng Huang, Cong Luo, KhaiHang Choong, Zexin Li, Yu Guo, Kit-Leong Cheong

**Affiliations:** a College of Marine Science, Guangxi Key Laboratory of Beibu Gulf Biodiversity Conservation, Beibu Gulf Ocean Development Research Center, Beibu Gulf University, Beibu Gulf Marine Ecological Environment Field Observation and Research Station of Guangxi, Qinzhou, Guangxi, China; b South China Sea Fisheries Research Institute, Chinese Academy of Fishery Sciences, Guangzhou 510300, China; c Guangdong Provincial Key Laboratory of Aquatic Product Processing and Safety, College of Food Science and Technology, Guangdong Ocean University, Zhanjiang 524088, China

**Keywords:** Bivalve aquaculture, Lipid, PUFA, EPA + DHA, Protein, EAA.

## Abstract

The rapid growth of the human population and urbanization has significantly increased the demand for animal proteins. However, expanding protein production from land-based farming, fisheries, and fish aquaculture faces challenges such as limited land and water resources, high carbon emissions, overfished stocks, and reliance on unsustainable fish meal and fish oil. Although many studies highlight bivalve aquaculture as a potential source of sustainable and high-quality proteins, there is limited quantitative data on the production of omega-3 long-chain polyunsaturated fatty acids (n-3 LC-PUFA) and essential amino acid (EAA) from global bivalve aquaculture. In this context, the present study aims to evaluate the current status of n-3 LC-PUFA and EAA production in global bivalve mariculture. The results of this study revealed that bivalves are a valuable source of high-quality animal protein, rich in essential amino acids (EAAs) and n-3 long-chain polyunsaturated fatty acids (LC-PUFA), such as EPA and DHA. Between 2018 and 2022, bivalve production increased by 7.1 % in wet weight, while the growth in crude protein, PUFA, EPA + DHA, and EEA yields was 4.4 %, 5.9 %, 6.5 %, and 3.1 %, respectively. Current production levels of EPA + DHA and EAAs from bivalve aquaculture are sufficient to meet the dietary needs of 78.68 million and 17.3 million healthy adults, respectively. Among different bivalve species, clams and scallops are the most efficient producers of EPA + DHA and EAAs, while oysters produce the least. This study provides a comprehensive overview of lipid and protein production in bivalve aquaculture and offers insights for future management strategies to support the industry's growth.

## Highlights

• Bivalves are a valuable source of high-quality animal protein.
• From 2018 to 2022, EPA + DHA and EAA yields from bivalves increased by 5.9 and 3.1 %.
• EPA + DHA and EAAs from bivalves meet the needs of 78.68 million and 17.3 million people.
• Clams and scallops are the most efficient in producing EPA + DHA and EAAs.
• Oysters produce the least EPA + DHA and EAAs.

## 1 Introduction

Food security is a global issue that requires immediate attention. In 2023, it was estimated that about 2.3 billion people (28.9 % of the world population) did not have regular excess to adequate nutrition, with 864 million people (10.7 % of the world population) experiencing severe food insecurity (Food and agriculture Organization of the United Nation (FAO), 2024a). Among essential nutrients, omega-3 long-chain polyunsaturated fatty acids (n-3 LC-PUFA), especially docosahexaenoic acid (DHA, 22:6n-3) and eicosapentaenoic acid (EPA, 20:5n-3), as well as essential amino acids (EAAs), play an important role in human growth and development. On the one hand, n-3 LC-PUFAs are crucial for fetal development, maintaining the health of eyes, hearth, and brain, and providing anti-inflammatory effects (Baker et al., 2020). Various major health organizations have proposed guidelines for EPA + DHA intake, with most recommending a daily minimum of 500 mg (Laukkanen et al., 2024; Vannice & Rasmussen, 2014). To date, the annual global production of EPA + DHA is estimated to be about 160 thousand tonnes (Glencross et al., 2024; Hamilton et al., 2020), while the demand for EPA + DHA has reached 1.48 million tonnes (500 mg/day × 365 days × 8.1 billion). As a result, deficiencies in n-3 LC-PUFAs have been reported globally, particularly in regions such as Central Europe, the United Kingdom, North America, the Middle East, and India (Stark et al., 2016).

On the other hand, amino acids play a critical role in the synthesis of enzymes, immune components, hormones, and neurotransmitters, as well as in energy production when carbohydrate and lipid intake is inadequate (Day et al., 2022; Henchion et al., 2017). Amino acids are categorized into two groups based on the body's ability to synthesize them: essential amino acids (EAAs) and non-essential amino acids. EAAs, including threonine (Thr), cystine (Cys), methionine (Met), valine (Val), isoleucine (Ile), leucine (Leu), phenylalanine (Phe), tryptophan (Trp), and lysine (Lys), cannot be synthesized by the body or are produced in insufficient quantities. Therefore, it is vital to obtain adequate amounts of EAAs through dietary intake. The recommended adequate intake of protein (Henchion et al., 2017) and the optimal EAA requirement for an adult male weighing 70 kg and 177 cm tall (National Institutes of Health, 2024) were 50 g/ capita/ day and 12.845 g/ capita/ day, respectively, with no upper limit defined.

As a result of urbanization and the drastic increase in global population, which has grown at a rate of 3 % per year, from 2.5 billion in 1950 to 8.1 billion in 2024 (World Population Clock, 2024), the gap of demand and supply for EPA + DHA and EAAs has widened. The growing demand and supply gap of EPA + DHA and EAAs is unlikely to be narrowed by increasing protein production from crops, livestock, and land-based aquaculture (freshwater farming), as land agriculture faces competition for limited land and water resources, with annual production currently showing a decreasing trend (Amundson et al., 2015; Costello et al., 2020; Foley, Defries, Asner, et al., 2005, Foley, Ramankutty, Rrauman, et al., 2011; Olsen, 2011). In fact, EPA and DHA are primarily sourced from marine environments, with terrestrial food contributing very little to global EPA and DHA production (Tocher et al., 2019). Although seafood is an excellent source of EPA + DHA and EAAs, over 90 % of fish stocks are overfished, and most marine fish aquaculture remains heavily dependent on unsustainable sources of fish meal and fish oil (Food and Agriculture Organization (FAO), 2018; Food and Agriculture Organization of the United Nation (FAO), 2024b, Glencross et al., 2024).

Marine bivalves are considered a high quality source of animal protein, very rich in n-3 LC-PUFA (Tan et al., 2021) and EAAs (Song et al., 2024). Unlike fish and crustaceans, bivalves are unfed species that do not require additional feed, relying entirely on phytoplankton and suspended organic particles from their surrounding water (Xu et al., 2024; Tan, Liu, et al., 2024). As a result, bivalve aquaculture has a low carbon footprint and has been promoted as an adaptation measure to address the challenges of climate change (Song, Luo, et al., 2024; Tan, Xu, et al., 2024). To date, bivalve aquaculture has expanded significantly, making up over 20 % of global aquaculture output (Food and Agriculture Organization of the United Nation (FAO), 2024b). However, the expansion of the bivalve aquaculture industry is uneven worldwide, with the majority of production concentrated in Asia, particularly in China, which accounts for over 85 % of global bivalve aquaculture production (Food and Agriculture Organization of the United Nation (FAO), 2024b). As demand for seafood continues to rise globally, the bivalve aquaculture industry is poised for further growth, driven by sustainability, innovation, and rising global demand for eco-friendly protein sources.

In this context, the current study was conducted to evaluate the performance of bivalve aquaculture in terms of lipid and protein production. The annual production of lipids and proteins from different bivalve groups was estimated based on the 2024 edition of the Food and Aquaculture Organization of the United Nation report (Food and Agriculture Organization of the United Nation (FAO), 2024b) and the average lipid and protein quality of bivalves, as reported by Tan et al. (2021) and Song, Luo, et al., 2024. The findings of this study can serve as a guide for formulating aquaculture management plans to further increase the production of EPA + DHA and EAAs, helping to narrow the gap between demand and supply for high quality animal protein.

## 2 Materials and methods

### 2.1 Data acquisition

The aquaculture production of major marine bivalve groups (oysters, clams, scallops, mussels, and razor clams) from 2018 to 2022 was extracted from the 2024 edition of the Food and Aquaculture Organization of the United Nation report (Food and Agriculture Organization of the United Nation (FAO), 2024b).

The average lipid nutritional quality was calculated based on the results of a meta-analysis on the fatty acid profile (Tan et al., 2021) and the amino acid profile (Song, Wang, et al., 2024) of global marine bivalves (Supplementary table 1).

### 2.2 Estimation of proteins and lipids from global bivalve aquaculture

The annual production of (a) lipids, (b) EPA + DHA, (c) PUFA, (d) proteins, (e) total AAs, and (f) EAAs was calculated using the following formulae:

(a) Lipid production = annual production (million tonne) X flesh content (%WW) X lipid content (% WW)
(b) EPA + DHA production = Annual production (million tonne) X flesh content (% WW) X EPA + DHA content (mg/g flesh) / 1000
(c) PUFA production = Production of EPA + DHA (thousand tonnes) X PUFA (%) / (EPA+ DHA (%))
(d) Protein production = Annual production (million tonnes) X flesh content (%WW) X protein content (g protein/ 100 g flesh)/ 10,000
(e) Total AA production = Protein production X AA content (g/100 g protein)/100
(f) EAA production = Protein production X EAA content (g/100 g protein)/ 100

### 2.3 Statistical analysis

All statistical analyses were performed using SPSS software (version 26), with significance difference set at *P* < 0.05 unless otherwise specified. All variables were presented as mean ± standard deviation (SD) and were assessed for normality and homogeneity of variance. One-way ANOVA, followed by Tukey's HSD post-hoc tests, was used to compare the yield, lipids, PUFA, EPA + DHA, proteins, AAs, and EAAs among different bivalves.

## 3 Results

### 3.1 Lipid and protein nutritional quality of marine bivalves

The average lipid and protein nutritional quality of marine bivalves was calculated and summarized in Table 1. In general, flesh content in marine bivalves ranged from 17.8 to 30.0 %, with scallops and razor clams having the highest flesh content (P < 0.05), while mussels and clams had the lowest (P < 0.05). In terms of lipid nutritional quality, the content of lipids, PUFA, and EPA + DHA in clams was significantly higher (P < 0.05) than in other bivalves, while it was significantly lower in scallops and razor clams. Regarding protein nutritional quality, the content of protein, AAs, and EAAs was highest (P < 0.05) in scallops and lowest (P < 0.05) in razor clams and oysters.

**Table 1:** Average lipid and protein nutritional quality of marine bivalves.

	Flesh content (%WW)	Lipid quality			Protein quality		
		Lipid (% WW)	PUFA (%)	EPA + DHA (mg/g flesh)	Protein content (g protein/100 g flesh)	Total AAs (g/100 g protein)	EEAs (g/100 g protein)
Oysters	17.78 ± 1.51^c^	1.61 ± 1.04^b^	45.06 ± 11.69^b^	3.47 ± 3.69^c^	11.60 ± 2.70^d^	38.75 ± 7.77^c^	14.00 ± 2.90^c^
Mussels	22.5 ± 1.92^b^	1.70 ± 1.08^a^	44.40 ± 15.47^b^	4.80 ± 2.68^b^	12.80 ± 0.20^c^	45.14 ± 9.00^b^	17.19 ± 3.95^b^
Scallops	30.00 ± 2.24^a^	1.20 ± 0.69^c^	37.70 ± 11.98^cd^	2.70 ± 1.54^d^	16.25 ± 1.45^a^	63.23 ± 5.59^a^	23.27 ± 3.15^a^
Clams	21.55 ± 1.89^b^	1.85 ± 0.80^a^	53.50 ± 9.90^a^	6.40 ± 4.05^a^	12.75 ± 2.05^c^	36.99 ± 9.11^c^	18.62 ± 4.88^b^
Razor clams	30.00 ± 2.35^a^	1.25 ± 0.92^c^	31.00 ± 17.70^d^	0.20 ± 0.02^e^	14.80 ± 2.30^b^	29.02 ± 7.33	13.17 ± 5.21^c^

Modified form Tan et al. (2021) and Song et al. (2024a).

### 3.2 Annual production of bivalves from global aquaculture from 2018 to 2022

**Fig. 1:**
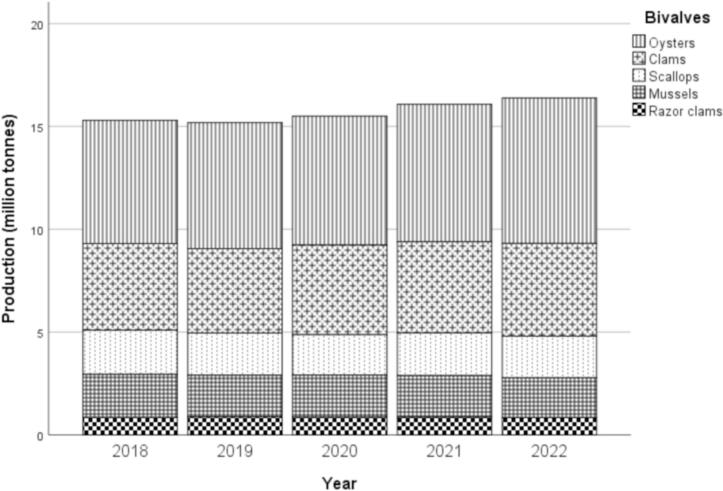
Annual production of bivalves from global aquaculture from 2018 to 2022.

The annual production of bivalves from global aquaculture increased by 7.1 %, from 15.3 million tonnes in 2018 to 16.4 million tonnes in 2022, with an average growth of 1.4 % per year (Fig. 1). The production of bivalves from aquaculture showed a descending order of oysters (41 %) > clams (25.5 %) > scallops (13.1 %) > mussels (12.9 %) > razor clams (5.5 %).

### 3.3 Annual production of lipids, PUFA, and EPA + DHA from global bivalve aquaculture from 2018 to 2022

**Fig. 2:**
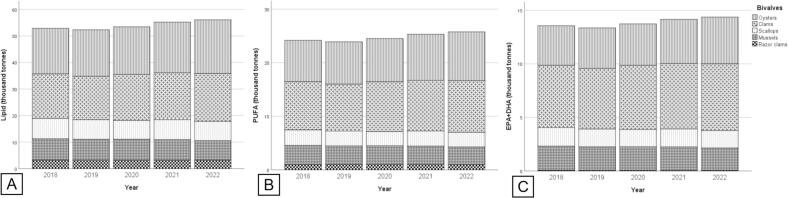
Annual production of (A) lipids, (B) PUFA, and (C) EPA + DHA from global bivalve aquaculture from 2018 to 2022.

The annual production of lipids and fatty acids from global aquaculture is illustrated in Fig. 2. The production of lipid increased by 6 %, from 52.87 thousand tonnes in 2018 into 56.07 thousand tonnes in 2022, with oysters (34.1 %) and clams (31.9 %) contributing the most, followed by mussels (14.3 %), scallops (13.7 %), and razor clams (6 %) (Fig. 2A).

From the perspective of PUFA, global production from bivalve aquaculture increased by 6.5 %, from 24.17 thousand tonnes in 2018 to 25.75 thousand tonnes in 2022, with an average growth of 1.3 % per year. The production of PUFA from bivalve aquaculture followed a descending order of oysters (33.5 %) > clams (37.3 %) > mussels (13.9 %) > scallops (11.3 %) > razor clams (4.0 %) (Fig. 2B). Among PUFA, the production of EPA + DHA from global bivalve aquaculture increased by 5.9 %, from 13.56 thousand tonnes in 2018 to 14.36 thousand tonnes in 2022, with clam aquaculture contributing the most (43.1 %), followed by oysters (28.7 %), mussels (15.8 %), scallops (12.0 %), and razor clams (0.4 %) (Fig. 2C).

### 3.4 Annual production of proteins, AAs, and EAAs from global bivalve aquaculture from 2018 to 2022

**Fig. 3:**
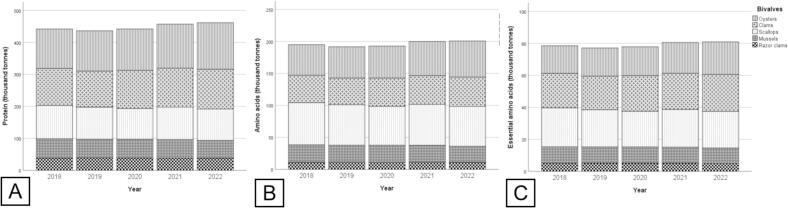
Annual production of (A) proteins, (B) AA, and (C) EAA from global bivalve aquaculture from 2018 to 2022.

The production of protein from global bivalve aquaculture increased by 4.4 %, from 442 thousand tonnes in 2018 to 461 thousand tonnes in 2022, with the aquaculture of oysters (29.6 %) contributed the most, followed by clams (26.5 %), scallops (22.3 %), mussels (13.0 %), and razor clams (8.5 %) (Fig. 3A).

In terms of amino acid production, it increased by 3 %, from 194.9 thousand tonnes in 2018 to 200.7 thousand tonnes in 2022, with the contribution of different bivalve groups in descending order of scallops (32.3 %) > oysters (26.2 %) > clams (22.4 %) > mussels (13.4 %) > razor clams (5.6 %) (Fig. 3B). Among AAs, the production of EAAs increased by 3.1 % from 78.5 thousand tonnes in 2018 to 81.0 thousand tonnes in 2022 (Fig. 3C). The production of EEAs from bivalve aquaculture followed a descending order of scallops (29.5 %) > clams (28.0 %) > oysters (23.5 %) > mussels (12.7 %) > razor clams (6.4 %).

## 4 Discussion

In general, animal protein is recognized as having higher nutritional value than plant protein, mainly attribute to animal protein has higher digestibility and bioavailabity to humans, as well as its higher levels of essential amino acids, which are more beneficial to human health (Day et al., 2022). Among animal proteins, those from marine animals have much higher nutritional quality, given their high digestibility (>90 %) and richness in amino acids, omega-3 LC-PUFAs, and other bioactive compounds with health benefits (Chasquibol et al., 2023; Song, Luo, et al., 2024). In recent decades, the drastic increase in the world population and average per capita income has significantly increased the demand for high quality animal proteins (Rahut et al., 2022). The uneven distribution of high-quality animal proteins toward high-income groups has caused serious food inequality issues, resulting in a greater need for food to meet the global demand for high quality animal protein (D'Odorico et al., 2019). In fact, more than one-third of the world's population (2.8 billion people) does not consume the healthy food required to meet minimal nutrient requirements, and more than 580 million people are chronically undernourished, especially in low-income countries, which account for over >70 % (Food and agriculture Organization of the United Nation (FAO), 2024a). Unfortunately, limited land area, overfishing, environmental degradation, and climate change have restricted the expansion of food production from land-based agriculture, fisheries, and fish aquaculture (MacLeod et al., 2020).

From the perspective of narrowing the gap between food supply and demand through the expansion of bivalve mariculture, an environmentally friendly and sustainable source of high quality, the production of bivalves from aquaculture has increased by 7.1 % from 2018 to 2022, a rate higher than the growth rate of the global human population (3.5 % increased from 7.73 billion in 2018 to 8.0 billion in 2022). This indicates the increasing role of bivalve mariculture in narrowing the demand and supply gap for high quality animal protein. In specific, the increase in the production rate of protein (4.4 %), PUFA (5.9 %), and EPA + DHA (6.5 %) from bivalve aquaculture between 2018 and 2022 was much higher than the growth rate of the global population (3.5 %), although the increased in EAA supply (3.1 %) still lagged behind. Despite the increasing production of EPA + DHA and EAAs from bivalve aquaculture, in 2022 (with a population of 8.0 billion), based on the recommended minimum intake of 500 mg of EPA + DHA per day (Laukkanen et al., 2024), an estimated adequate protein consumption rate of 50 g/ capita/ day (Henchion et al., 2017), and an EAA requirement of 12.845 g/ capita/ day for an adult male weighing 70 kg and 177 cm tall (National Institutes of Health, 2024), the current production of EPA + DHA (14.36 thousand tonnes), protein (462 thousand tonnes), and EAAs (81 thousand tonnes) meets only the requirements of 0.98 % (78.68 millions), 0.31 % (25 million), and 0.22 % (17.3 million) of the global population.

It is estimated that the current global production of EPA + DHA is about 160 thousand tonnes per year (meeting the recommended minimum EPA + DHA intake for 11 % of global population), with global aquaculture contributing a net 58 thousand tonnes of EPA + DHA production per year (Glencross et al., 2024; Hamilton et al., 2020). It is worth noting that the current production of EPA + DHA from global bivalve aquaculture accounts for about 25 % of the EPA + DHA produced by global aquaculture, despite bivalve aquaculture representing only 8.86 % of the total production of aquatic animals from aquaculture (FAO, 2024b), indicating that bivalve aquaculture is highly efficient in producing EPA + DHA. Among bivalve species, the aquaculture of clams is the most effective for PUFA and EPA + DHA production. Although clam aquaculture accounts for only 26 % of global aquaculture production, it produces 37 % of PUFA and 43 % of EPA + DHA. Conversely, the aquaculture of oysters and razor clams is less favorable for EPA + DHA production, with oysters and razor clam aquaculture contributing only 28.7 % and 0.4 %, despite their much higher shares in global aquaculture production of 41 % and 5.5 %, respectively. Therefore, expanding the aquaculture of clams would significantly boost the production of EPA + DHA.

From the perspective of EAA production, based on the estimation of crude protein production from terrestrial animals (76 million tonnes) and aquaculture animals (6.815 million tonnes) (Boyd et al., 2022), the production of crude protein from bivalve aquaculture (0.442 million tonnes) represent only 0.57 % of terrestrial animals and 6.49 % of aquaculture animals. Among bivalves, scallop aquaculture is the best choice for EAA production, contributing 29.5 % of the total EAAs from global bivalve aquaculture, despite accounting for only 13.1 % of total global bivalve production. However, the aquaculture of oysters is less favorable for EAA production, contributing only 23.5 % of EAAs from bivalve aquaculture despite representing over 40 % of total aquaculture yield. Therefore, to increase the production of EAAs and EPA + DHA, the expansion of scallop and clam aquaculture should be prioritized in future aquaculture development plans, whereas the aquaculture of oysters appears less effective in producing EAAs and EPA + DHA, despite being the most produced bivalves (41 %).

It is worth noting that, except for China, most coastal nations currently use only less than 1 % of their sea area suitable for bivalve aquaculture, and there are still more than 1.5 million km^2^ of ocean suitable for bivalve aquaculture (Gentry et al., 2017). If these areas are utilized for bivalve aquaculture, it could easily increase the production of bivalves from aquaculture by 100-fold. The main challenge for the development of bivalve aquaculture in most coastal nations is their heavy reliance on inconsistent seed supplies from the wild (Symonds et al., 2019). In fact, the key to success in China's bivalve aquaculture industry has been their significant technical breakthrough in producing bivalve seed in hatcheries (Wijsman et al., n.d.). Therefore, other coastal nations should also focus on developing their own bivalve hatcheries to support the expansion of bivalve aquaculture.

Caution though, this study uses only broad classifications of bivalves, such as scallops, clams, and razor clams, in estimating the total production of EAAs and n-3 LC_PUFA, which may overestimate or underestimate the actual contribution of different bivalve species to the supply of these macronutrients. This is because bivalves have limited LC-PUFA biosynthetic abilities, and their LC-PUFA content highly depends on their diets. Therefore, even within the same species, the LC-PUFA content in bivalves can vary significantly. Furture estimations of the production of these macronutrients in specific bivalve species are highly recommended to verify the results of this article. In addition, food processing and storage also are important factors affecting the quality of macronutrients (Tan, Huang, et al., 2023; Tan, Lim, & Peng, 2023; Tan, Lu, et al., 2023; Yan et al., 2024) and should be consider in future studies.

## 5 Conclusions

In conclusion, bivalves are an important source of high quality animal protein rich in EAAs and n-3 LC-PUFA (EPA and DHA). From 2018 to 2022, the production of bivalves increased by 7.1 % by wet weight, while the yield of crude protein, PUFAs, EPA + DHA, and EAAs increased by 4.4 %, 5.9 %, 6.5 %, and 3.1 %, respectively. The current production of EPA + DHA and EAAs meets the requirements of 78.68 millions and 17.3 million health adults, respectively. Among bivalves, clams and scallops are the most effective at producing EPA + DHA and EAAs, respectively, while oyster aquaculture produces the least. The results of this study not only provide an overview of the current status of lipid and protein production from bivalve aquaculture but also offer guidance for the development of management plans to be implemented in the future expansion of bivalve aquaculture.

## CRediT authorship contribution statement

**Karsoon Tan:** Writing – review & editing, Writing – original draft, Visualization, Validation, Supervision, Software, Resources, Project administration, Methodology, Investigation, Funding acquisition, Formal analysis, Data curation, Conceptualization. **Peng Xu:** Visualization. **Leiheng Huang:** Project administration. **Cong Luo:** Methodology. **KhaiHang Choong:** Validation. **Zexin Li:** Project administration. **Yu Guo:** Funding acquisition, Resources, Validation, Writing – review & editing. **Kit-Leong Cheong:** Supervision.

## Declaration of competing interest

The authors declare that they have no known competing financial interests or personal relationships that could have appeared to influence the work reported in this paper.

## Data Availability

The data that support the findings of this study are available from the corresponding author upon reasonable request.

## Appendix A Supplementary data

Supplementary material Image 1
