# Supplementary material for: Quantitative evaluation of essential amino acids and omega-3 long-chain polyunsaturated fatty acids from global marine bivalve aquaculture
Source: Food Chem X. 2025 Jan 23;25:102181. doi: 10.1016/j.fochx.2025.102181 (PMC11803897; doi:10.1016/j.fochx.2025.102181)
Supplement: Supplementary file 1 — Supplementary material [file mmc1.docx]

Supplementary table 1: Summary of genus of the marine bivalves involved in this study

| **Bivalve** | **Genus** | **References** |
| --- | --- | --- |
| Clams | *Arca* | Dupcic et al., 2014; Biandolino et al., 2019 |
|  | *Atrina* | Lee et al., 2021 |
|  | *Bonax* | Abirami et al., 2015 |
|  | *Chamelea* | Orban et al., 2007; Bityutskaya et al., 2021 |
|  | *Donaxtrunculus* | Bityutskaya et al., 2021 |
|  | *Ensis* | Baptista et al., 2014 |
|  | *Laternula* | Ahn et al., 2000 |
|  | *Limaria* | Biandolino et al., 2019 |
|  | *Macoma* | Misra et al., 1985; Wenne and Polak, 1989 |
|  | *Macruronus* | Mclean and Bulling, 2005 |
|  | *Mactra* | Tabakaeva and Tabakaev, 2018; Chetoui et al., 2019 |
|  | *Megangulus* | Kawashima et al., 2003 |
|  | *Meretrix* | Chen et al., 2012; Moniruzzaman et al., 2021 |
|  | *Paphia* | Chen et al., 2012 |
|  | *Ruditapes* | Ojea et al., 2004 |
|  | *Sinonovacula* | Wu et al., 2019 |
|  | *Solen* | Wu et al., 2019; Biandolino et al., 2019; Trisyani and Yusan, 2020 |
|  | *Spisula* | Tabakaeva and Tabakaev, 2017 |
|  | *Tegillarca* | Nguyen et al., 2017 |
|  | *Venerupi* | Ruano et al., 2012 |
|  |  |  |
| Oyster | *Crassostrea* | Pernet et al., 2007; Dridi et al., 2007; Ruano et al., 2012; Chen et al., 2012; Ricardo et al., 2015; Asha et al., 2014; Chakraborty et al., 2016; Peralta et al., 2018; Qin et al., 2018; Zhu et al., 2018; Huang et al., 2019; Tan et al., 2020; Moniruzzaman et al., 2021 |
|  | *Hyotissa* | Hong et al., 2022 |
|  | *Ostrea* | Ezgeta-Balic et al., 2012; Aziz et al., 2013; Biandolino et al., 2019 |
|  | *Saccostrea* | Mclean and Bulling, 2005; Moniruzzaman et al., 2021 |
|  |  |  |
| Cockles | *Anadara* | Aziz et al., 2013; Bityutskaya et al., 2021; Moniruzzaman et al., 2021 |
|  | *Fulvia* | Liu et al., 2013 |
|  | *Gafrarium* | Babu et al., 2012 |
|  | *Venus* | Miletic et al., 1991 |
|  |  |  |
| Scallops | *Argopecten* | Chasquibol et al., 2023 |
|  | *Chlamys* | Tan et al., 2019 |
|  | *Flexopecten* | Telahigue et al., 2013; Prato et al., 2019; Biandolino et al., 2019 |
|  | *Mimachlamys* | Biandolino et al., 2019 |
|  | *Pecten* | Manthey-Karl et al., 2015 |
|  | *Placopecten* | Manthey-Karl et al., 2015 |
|  |  |  |
| Mussels | *Batissa* | Jamaluddin et al., 2016 |
|  | *Modiolus* | Biandolino et al., 2019 |
|  | *Mytilus* | Miletic et al., 1991; Freites et al., 2002; Lin et al., 2003; Mclean and Bulling, 2005; Sengor et al., 2008; Prato et al., 2010; Redmond et al., 2010; Ezgeta-Balic et al., 2012; Ruano et al., 2012; Dernekbası et al., 2015; Merdzhanova et al., 2017; Biandolino et al., 2019 |
|  | *Perna* | Mclean and Bulling, 2005; Taylor et al., 2006; Chakraborty et al., 2011 |

**References**

Abirami P, Giji S, Mohan K, et al. Proximate composition, amino acid profile and mineral content of beach clam (*Bonax cuneatus*). *Fishery Technology* 2015; 52: 191-193.

Ahn IY, Cho KW, Choi KS, et al. Lipid content and composition of the *Antartic Lamellibranch*, *Laternula elliptica* (King & Broderip) (Anomalodesmata: Laternulidae), in King George Island during an austral summer. *Polar Biology* 2000; 23 (1): 24-33.

Asha KK, Anandan R, Mathew S, et al. Biochemical profile of oyster *Crassostrea madrasensis* and its nutritional attributes. *Egyptian Journal of Aquatic Research* 2014; 40: 35-41.

Aziz NA, Azlan A, Ismail A, et al. Quantitative determination of fatty acids in marine fish and shellfish from warm water of Straits of Malacca for Nutraceutical purposes. *BioMed Research International* 2013: 1–12.

Babu A, Venkatesan V, Rajagopal S. Biochemical composition of different body parts of *Gafrarium tumidum* (Roding, 1798) from Mandapam, South East Coast of India. *African Journal of Biotechnology* 2012; 11: 1700-1704.

Baptista M, Repolho T, Maulvault AL, et al. Temporal dynamics of animo and fatty acid composition in the razor clam *Ensis siliqua* (Mollusca: Bivalvia). *Helgoland Marine Research* 2014; 68(4): 465-482.

Biandolino F, Di Leo A, Parlapiano I, et al. Nutritional quality of edible marine bivalves from the Southern Coast of Italy, Mediterranean Sea. *Polish Journal of Food and Nutrition Sciences* 2019; 69(1): 71-81.

Bityutskaya OE, Donchenko LV, Moshenec KI. Analysis of technical and chemical characteristic as well as the nutritional value of clams from the Sea of Azov. *IOP Conference Series: Earth and Environmental Science* 2021; 640: 032045.

Chakraborty K, Chakkalakal SJ, Joseph D, et al. Nutritional composition of edible oysters (*Crassostrea madrasensis* L.) from the southwest coast of India. *Journal of Aquatic Food Product Technology* 2016; 25(8): 1172-1189.

Chakraborty K, Vijayagopal P, Asokan PK, et al. Green mussel (*Perna viridis* L.) as healthy food, and as a nutraceutical supplement. *Central Marine Fisheries Research Institute* 2011; 89-94.

Chasquibol N, Gonzales BF, Alarcón R, et al. Optimisation and characterisation of the protein hydrolysate of scallops (*Argopecten purpuratus*) visceral by-product. *Foods (Basel, Switzerland)* 2023; 12(10): 2003.

Chen DW, Su J, Liu DM, et al. Amino acid profile of bivalve molluscs from Beibu Gulf, China. *Journal of Aquatic Food Product Technology* 2012; 21(4): 369-379.

Chetoui I, Rabeh I, Bejaoui S, et al. First seasonal investigation of the fatty acid composition in three organs of the Tunisian bivalve Mactra stultorum. *Grasas Y Aceites* 2019; 70(1): e291.

Dernekbası S, Oksuz A, Celik MY, et al. The fatty acid composition of cultured mussels (*Mytilus galloprovincialis* Lamarck 1819) in offshore longline system in the Black Sea. *Journal of Aquaculture and Marine Biology* 2015; 2(6): 00049.

Dridi S, Salah Romdhane M, Elcafsi M. Seasonal variation in weight and biochemical composition of the Pacific oyster, *Crassostrea gigas* in relation to the gametogenic cycle and environmental conditions of the Bizert lagoon, Tunisia. *Aquaculture* 2007; 263(1-4): 238-248.

Dupcic Radic I, Cari M,Najdek CM, et al. Biochemical and fatty acid composition of *Arca noae* (Bivalvia: Arcidae) from the Mali Ston Bay, Adriatic Sea. *Mediterranean Marine Science* 2014; 15(3): 520-531.

Ezgeta-Balic D, Najdek M, Peharda M, et al Seasonal fatty acid profile analysis to trace origin of food sources of four commercially important bivalves. *Aquaculture* 2012; 334-337: 89-100.

Freites L, Fernandez-Reiriz MJ, Labarta U. Fatty acid profiles of *Mytilus galloprovincialis* (Lmk) mussel of subtidal and rocky shore origin. *Comparative Biochemistry and Physiology Part B: Biochemistry and Molecular Biology* 2002; 132 (2): 453-461.

Hong HK, Koo JH, Ko JC, et al. Proximate composition, amino acids, and fatty acids in the adductor muscle of the giant honeycomb oyster *Hyotissa hyotis* (Linnaeus, 1758) from Jeju Island, Korea. *Journal of Shellfish Research* 2022; 4(1): 101-107.

Huang YQ, Yang FM, Qin XM, et al. Chemical composition and characteristic odorans of oyster (*Crassostrea hongkongensis*) from different culture areas. *Food Science* 2019; 40(14): 236-242. (in Chinese with English abstract)

Jamaluddin, Mappiratu, Septiawan et al. Analysis of fatty acid and amino acid profile of “METI” mussels (*Batissa violacea* L. von Lamarck, 1818) in La’a River of Petasia district north Morowali Regency. *Rasayan Journal of Chemistry* 2016; 9(4): 673-679.

Kawashima H, M. Ohnishi. Fatty acid compositions of various tissue lipids in the marine bivalves, *Megangulus venulosus* and *Megangulus zyonoensis*, from coastal waters of Hokkaido, Northern Japan. *Journal of Oleo Science* 2003; 52(6):309-315.

Lee HJ, Roy VC, Ho TC, et al. Amino acid profiles and biopotentiality of hydrolysates obtained from comb penshell (*Atrina pectinata*) viscera using subcritical water hydrolysis. *Marine Drugs* 2021; 19: 137.

Lin H, Jiang J, Xue CH, et al. Seasonal changes in phospholipids of mussel (*Mytillus edulis* Linne). *Journal of the Science of Food and Agriculture* 2003; 83(2): 133-135.

Liu W, Li Q, Kong L. Reproductive cycle and seasonal variations in lipid content and fatty acid composition in gonad of the cockle *Fulvia mutica* in relation to temperature and food. *Journal of Ocean University of China* 2013; 12(3): 427-433.

Manthey-Karl M, Lehmann I, Ostermeyer U, et al. Meat composition and quality assessment of King Scallops (*Pecten maximus*) and frozen Atlantic Sea scallops (*Placopecten magellanicus*) on a retail level. Foods 2015; 4(4): 524-546.

Mclean CH, Bulling KR. Differences in lipid profile of New Zealand marine species over four seasons. *Journal of Food Lipids* 2005; 12(4): 313-326.

Merdzhanova A, Dobreva DA, Panayotova V. Assessment of proximate and bioactive lipid composition of Black Sea Mussels (*M. galloprovincialis*) from Bulgaria. Biological Resources of Water Sajal Ray, IntechOpen 2017; doi:10.5772/intechopen.71909.

Miletic I, Miric M, Lalic Z, et al. Composition of lipids and proteins of several species of molluscs, marine and terrestrial, from the Adriatic Sea and Serbia. *Food Chemistry* 1991; 41(3): 303-308.

Misra S, Ghosh MK, Choudhury A, et al. Fatty acids from *Macoma* sp. of bivalve mollusk. *Journal of the Science of Food and Agriculture* 1985; 36(11): 1193-1196.

Moniruzzaman M, Sku S, Chowdhury P, et al. Nutritional evaluation of some economically important marine and freshwater mollusc species of Bangladesh. *Heliyon* 2021; 7: 07088.

Nguyen TT, Choi YJ, Rohmah Z, et al. Seasonal variations of nutritional components in cockles (*Tegillarca granosa*) processed from the Southern Coast of Korea. *Cogent Food and Agriculture* 2017; 3(1): 1360102.

Ojea J, Pazos AJ, Martınez D, et al. Seasonal variation in weight and biochemical composition of the tissues of *Ruditapes decussatus* in relation to the gametogenic cycle. *Aquaculture* 2004; 238(1-4): 451-468.

Orban E, Di Lena G, Nevigato T, et al. Nutritional and commercial quality of the striped venus clam, *Chamelea gallina*, from the Adriatic sea. *Food Chemistry* 2007; 101(3): 1063-1070.

Peralta EM, Monaya KJM, Simora RMC, et al. Chemical composition and antioxidant properties of Philippine oyster (*Crassostrea iredalei*) residue. *Philippine Journal of Natural Science* 2018; 22(1): 19-27.

Pernet F, Gauthier-Clec S, Mayrand E. Change in lipid composition in eastern oyster (*Crassostrea virginica* Gmelin) exposed to constant or fluctuating temperature regimes. *Comparative Biochemistry and Physiology Part B: Biochemistry and Molecular Biology* 2007; 147(3): 557-565.

Prato E, Biandolino F, Parlapiano I, et al. Seasonal changes of commercial traits, proximate and fatty acid compositions of the scallop *Flexopecten glaber* from the Mediterranean Sea (Southern Italy). *PeerJ* 2019; 7: 5810.

Prato, E., A. Danieli, M. Maffia, and F. Biandolino. Lipid and fatty acid compositions of *Mytilus galloprovincialis* cultured in the Mar Grande of Taranto (Southern Italy): Feeding strategies and trophic relationships. Zoological Studies 2010; 49(2): 211-219.

Qin Y, Zhang Y, Ma H, et al. Comparison of the biochemical composition and nutritional quality between diploid and triploid Hong Kong oysters, *Crassostrea hongkongensis*. *Frontiers in Physiology* 2018; 9:1674.

Redmond KJ, Magnesen T, Hansen PK, et al. Stable isotopes and fatty acids as tracers of the assimilation of salmon fish feed in blue mussels (*Mytilus edulis*). *Aquaculture* 2010; 298(3-4): 202-210.

Ricardo F, Pimentel T, Moreira ASP, et al. Potential use of fatty acid profiles of the adductor muscle of cockles (*Cerastoderma edule*) for traceability of collection site. *Scienctific Reports* 2015; 5: 11125.

Ruano F, Ramos P, Quaresma M. Evolution of fatty acid profile and condition index in mollusk bivalves submitted to different depuration periods. *Revista Portuguesa de Ciencias Veterinarias* 2012; 111(581–582): 75-84.

Sengor GF, Gun HY, Kalafatoglu H. Determination of the amino acid and chemical composition of canned smoked mussels (*Mytilus galloprovincialis*, L.). *Turkish Journal of Veterinary and Animal Sciences* 2008; 32(1): 1-5.

Tabakaeva OV, Tabakaev AV. Lipids, vinyl alcohols, and fatty acids from soft tissues of the bivalve mollusc Mactra chinensis. *Chemistry of Natural Compounds* 2018; 54(2): 223-227.

Tabakaeva OV, Tabakaev AV. Lipids and fatty acids from soft tissues of the bivalves mollusc *Spisula sachalinensis*. *Chemistry of Natural Compounds* 2017; 53(1): 16-20.

Tan KS, Liu HX, Ye T, et al. Growth, survival and lipid composition of *Crassostrea gigas*, *C. angulata* and their reciprocal hybrids cultured in southern China. *Aquaculture* 2020; 516: 734524.

Tan KS, Leng XM, Zhao Y, et al. Amino acid variations in polymorphic noble scallops, *Chlamys nobilis*. *Journal of Food Processing and Preservation* 2019; 00: 14262.

Taylor AG, Savage C. Fatty acid composition of New Zealand green-lipped mussels, *Perna canaliculus*: Implications for harvesting for n-3 extracts. *Aquaculture* 2006; 26*1*(1): 430-439.

Telahigue K, Hajji T, Rabeh I, et al. The effect of starvation on the biochemical composition of the digestion gland, the gonad and the adductor muscle of the scallop *Flexopecten glaber*. *Food and Nutritional Science* 2013; 4: 405-413.

Trisyani N, Yusan LY. Proximate analysis and amino acid profile in fresh meat, meat meal and shell meal of bamboo clam *Solen* sp. from Kwanyar Coast, Bangkalan Madura, Indonesia. *AACL Bioflux* 2020; 13(5): 2921-2927.

Wenne R, Polak L. Lipid composition and storage in the tissues of the bivalve, *Macoma balthica*. *Biochemical Systematics and Ecology* 1989; 17(7-8): 583-587.

Wu Z, Zhou D, Zhao M, et al. Lipid profile and glycerophospholipid molecular species in two species of edible razor clams *Sinonovacula constricta* and *Solen gouldi*. *Lipids* 2019; 54(5): 3470-356.

Zhu YJ, Li Q, Yu H, et al. Biochemical composition and nutritional value of different shell color strains of Pacific oyster *Crassostrea gigas*. *Journal of Ocean University of China* 2018; 17(4): 897-904.
